# *Theileria annulata* Cyclophilin1 (TaCyp1) Interacts With Host Cell MED21

**DOI:** 10.3389/fmicb.2018.02973

**Published:** 2018-12-03

**Authors:** Shuaiyang Zhao, Junlong Liu, Guiquan Guan, Aihong Liu, Youquan Li, Hong Yin, Jianxun Luo

**Affiliations:** ^1^State Key Laboratory of Veterinary Etiological Biology, Key Laboratory of Veterinary Parasitology of Gansu Province, Lanzhou Veterinary Research Institute, Chinese Academy of Agricultural Sciences, Lanzhou, China; ^2^Jiangsu Co-innovation Center for Prevention and Control of Important Animal Infectious Diseases and Zoonoses, Yangzhou, China

**Keywords:** *Theileria annulata*, schizont, transformation, cyclophilins, MED21, NF-κB, interaction

## Abstract

Host cells infected by *Theileria annulata* schizonts show the character of permanent proliferation *in vitro*, also named transformation. To explore the molecular mechanism a *T. annulata* Cyp1 (TaCyp1) protein potentially involved in regulating cell transformation was used as bait to screen for its interacting proteins by yeast-two-hybrid assay. Additional GST-pull down experiments confirmed that only MED21 specifically interacted with TaCyp1. Moreover, the distribution of TaCyp1 around *T. annulata* schizonts facilitated interaction with host cell MED21. As a component of mediator complex, MED21 is normally involved in regulating the transcription of nearly all RNA polymerase II-dependent genes. Therefore, to explore its influence on NF-κB signaling MED21 RNA interference and parasite killing with BW720c treatment were performed. Knock down of MED21 resulted in a significant decrease in NF-κB1/2 mRNA expressions, but no significant change in P105, P52 levels, nor detectable alteration in levels of phosphorylated IκBα/β. By contrast, BW720c treatment induced an obvious decrease in the phosphorylation status of P52 and IκBα/β, but no obvious change in that of P105. This suggests that BW720c-induced parasite death had a significant negative influence on NF-κB signaling, whereas knock down of MED21 had no obvious effect on NF-κB signaling. Characterization of TaCyp1 provides information on the function of parasite cyclophilins and leads to a better understanding of the interactions between *T. annulata* and its host leukocytes.

## Introduction

*Theileria annulata* is a tick-borne protozoan parasite mainly distributed in North Africa, Southern Europe, India, the Middle East, and Asia (Bilgic et al., 2010). As the causative agents of tropical theileriosis it infects bovine monocytes/macrophages, B lymphocytes and erythrocytes resulting in lymphadenopathy, hyperthermia, anemia and emaciation (Glass et al., 1989). After sporozoite invasion *T. annulata* develops into macro-schizonts and infected leukocytes enter into transformed state showing for example, permanent proliferation *in vitro* (Shaw, 2003). However, the transformed state can be reversed by treating infected leukocytes with the anti-parasite drug Buparvaquone720c (BW720c) (Heussler et al., 2002). Cyclophilins (Cyps) also known as immunophilins are a family of ubiquitous proteins present in the cytosol of all prokaryotic and eukaryotic cells. Due to their peptidyl-prolyl isomerase (PPIase) activity they catalyze the *cis* to *trans* conversion of proline-containing peptides that facilitates protein folding (Wang and Heitman, 2005; Naoumov, 2014). In general, cyclophilins have a common 109 amino acid cyclophilin-like domain (CLD) and additional domains unique to each member. Among all family members CypA is most abundantly distributed in the cytosol (Dornan et al., 2003), where it’s involved in protein folding, trafficking, signal transduction and cell activation (Nigro et al., 2013). Among parasites a *Trichomonas vaginalis* cyclophilin TvCyp1 is involved in the nuclear translocation of Myb1 and Myb3, which regulate transcription of the adhesion protein ap65-1 (Hsu et al., 2014; Chu et al., 2018). Floudas et al. (2017) found the recombinant *Schistosoma mansoni* CypA was capable of modulating bone marrow derived dendritic cell (BMDC) and T cell responses *in vitro*. Bustos et al. (2017) found overexpression of *Trypanosoma cruzi* CyPD potentially enhanced the loss of mitochondrial membrane potential and cell viability when the parasite was exposed to a hydrogen peroxide stimulus. Ibrahim et al. (2014) found *Toxoplasma gondii* cyclophilin18 regulated host cell migration and enhanced parasite dissemination in a CCR5-independent manner. Maeda et al. (2013) found that recombinant *Haemaphysalis longicornis* cyclophilin A (HlCyPA) significantly inhibited the growth of *Babesia bovis* and *B. bigemina* *in vitro*, indicating that HlCyPA potentially regulated *Babesia* growth in *H. longicornis* ticks. Marín-Menéndez et al. (2012) found two *Plasmodium falciparum* cyclophilin proteins had isomerase activity and displayed chaperone function. Kameyama et al. (2012) demonstrated that *Neospora caninum* cyclophilin was distributed in tachyzoites around brain lesions and caused the migration of murine and bovine cells in cysteine-cysteine chemokine receptor 5-dependent way. For *T. annulata* cyclophilins, their role in cell transformation is almost unknown.

Without the restriction of a parasitophorous vacuole, *T. annulata* schizonts are exposed in the cytoplasm with the opportunity to interfere with host cell signaling pathways (Shiels et al., 2006), such as JNK (Galley et al., 1997), c-Myc (Dessauge et al., 2005), Notch (Chaussepied et al., 2006), protein kinase-A (PKA) (Guergnon et al., 2006), and NF-κB pathways (Heussler et al., 2006). To date, some proteins from *T. annulata* schizonts have been shown to regulate host cell proliferation and/or survival. For instance, the AT hook domain-rich proteins TashAT1/2/3 and SuAT1 have been demonstrated to translocate to the host cell nucleus and regulated cell proliferation (Swan et al., 1999, 2001; Shiels et al., 2004). The *T. annulata* secreted protein TaSE has been shown to interact with α-tubulin and potentially regulate cell mitosis (Schneider et al., 2007). Similar to TaSE GPI-anchored gp34 was able to induce cytokinetic defects and cause accumulation of binucleated cells indicating gp34 to interferes with cell division (Xue et al., 2010). Through interaction with the microtubule network TaSP was found to participate in cell proliferation (Seitzer et al., 2010). Recently, a polymorphic membrane protein, P104 was shown to recruit and activate end-binding protein1 to regulate host cell microtubule network dynamics (Woods et al., 2013). In 2015 a prolyl-isomerase, TaPIN1 was shown to induce the degradation of the ubiquitin ligase FBW7 stabilizing c-JUN to sustain cell transformation (Marsolier et al., 2015). With the accumulation of genomics data (Pain et al., 2005) a *T. annulata* cyclophilin (TaCyp1, accession no: XM_949388.1) was identified and found to potentially regulate host cell transformation (Marsolier et al., 2015). In the present study, TaCyp1 was used as bait protein to screen for interaction proteins by yeast-two-hybrid assay. Sub-cellular location experiments were also performed to determine its distribution in host cells. Subsequently, GST-pull down confirmed a host protein that specifically interacts with TaCy1. Previous work has shown that the nuclear transcription factor NF-κB is involved in cell proliferation, differentiation, adhesion, stress and inflammation, and often activated in tumor cells (Gilmore, 2006; Hayden and Ghosh, 2008, 2011; Vallabhapurapu and Karin, 2009). In *T. annulata* infected cells NF-κB is activated to regulate cell proliferation preventing host cells from undergoing apoptosis (Heussler et al., 1999; Dobbelaere and Rottenberg, 2003). Schmuckli-Maurer et al. (2010) found that *T. annulata*-dependent IKK signalosomes and actin were involved in regulating NF-κB activation in infected cells. To explore the influence on NF-κB signaling pathway of host proteins interacting with TaCyp1 we examined the expression of NF-κB1/2 and the phosphorylation status of IκBα/β following knockdown of TaCyp1 by RNA interference (RNAi) and drug-induced parasite death following BW720c treatment. Characterization of TaCyp1 will provide more information on the function of parasite cyclophilins and will lead to a better understanding of interactions between *T. annulata* and its host cell.

## Materials and Methods

### Cell Culture

The *T. annulata* schizont-infected cell line (TaNM1) was obtained and conserved by the Vector and Vector-borne Disease (VVBD) laboratory, Lanzhou Veterinary Research Institute (LVRI), China. Cells were maintained in RPMI 1640 (Gibco) supplemented with 10% fetal bovine serum (Gibco) and 100 mg/ml penicillin/streptomycin at 37°C in a humidified 5% CO_2_ incubator.

### Construction of Yeast Two-Hybrid cDNA Library of Bovine B Cells

Separation of B cell from bovine peripheral blood mononuclear cells (PBMCs) and construction of a yeast two-hybrid cDNA library have been previously described (Zhao et al., 2017). Briefly, 2 × 10^7^ B cells with purity of 95.3% were obtained and used for cDNA library construction and library titer was 2 × 10^6^ cfu with the size of inserted fragments varying from 750 to 3500 bp.

### Bait Plasmid Expression in Yeast Cells

**FIGURE 1:**
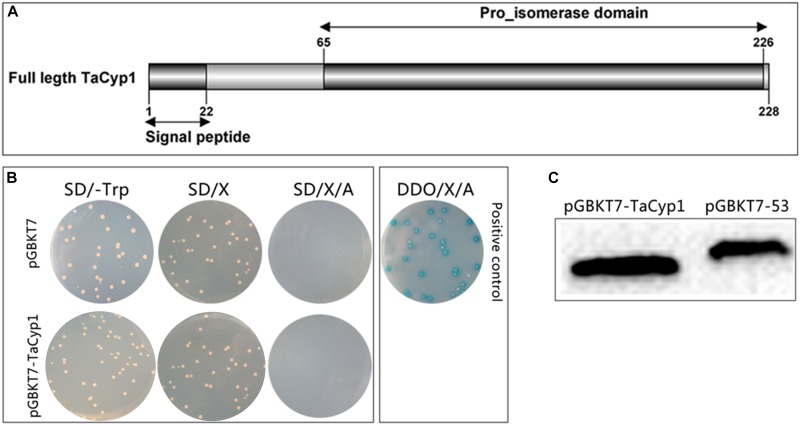
Gene structure, expression, auto-activation, and toxicity tests for pGBKT7-TaCyp1. **(A)** Gene structure of TaCyp1, the whole length (aa1-228) of TaCyp1 was used in yeast-two-hybrid screening. **(B)** Western blot analysis of total proteins extracted from Y2HGold cells. TaCyp1 bait protein and positive control were 45 and 57 kDa in size, respectively. **(C)** Analysis of auto-activation and toxicity activity of pGBKT7-TaCyp1 bait plasmid in Y2HGold cells. The pGBKT7- TaCyp1 bait and pGBKT7 plasmids were transformed to Y2HGold cells and the transformants were grown on SD, SD/X, and SD/X/A agar plates, respectively; the co-transformants containing pGADT7-T and pGBKT7-53 were grown on DDO/X/A agar plates as positive control.

Total RNA of TaNM1 cells was extracted by using TRIzol Reagent and reverse transcribed into 1st strand cDNA. A 684 bp fragment of TaCyp1 gene encoding 228 amino acid residues (accession no.: XM_949388.1) (Figure 1A) was amplified from the cDNA and cloned into pGBKT7 plasmid between restriction sites *EcoRI* and *BamHI* using oligonucleotides: forward, CCGGAATTCATGCATCTTAGACAAAATATC, reverse, CGC GGATCCCAATAATTCTCCACAGTCCTC. Following the protocols of the Yeastmaker^TM^ Yeast Transformation System 2 kit (Clontech, United States) both pGBKT7-TaCyp1 and pGBKT7-53 (positive control) were transformed into the yeast strain Y2HGold. Total proteins were extracted from yeast cells and detected using mouse anti-Myc tag McAb (1/1000, Proteintech, United States) to detect TaCyp1 expression (Zhao et al., 2017).

### Auto-Activation and Toxicity Tests of Bait Plasmid

The protocol has been described previously (Zhao et al., 2017) and according to white colonies on SD/-Trp and SD/-Trp/X plates, and absence of colony growth on SD/-Trp/X/A plates, the bait plasmid was confirmed to lack auto-activation. Moreover, similar colonies size between bait and pGBKT7 plasmids indicated no toxicity indicating that expression of the bait plasmid had no auto-activation and toxicity and could be used in the yeast-two-hybrid screen.

### Yeast-Two-Hybrid Screen and Sequences Analysis

To screen for host interacting proteins TaCyp1 bait and prey plasmids were co-transfected into Y2HGold, and screened as previously described (Zhao et al., 2017). The blue colonies on QDO/X/A agar plates were confirmed as positive hits. To reduce the number of false positives prey plasmids of the putatively positive colonies were rescued and co-transformed into Y2HGold with TaCyp1 bait plasmid, respectively. The co-transformant blue colonies were considered true positive hits.

Using primers of pGADT7-F/R the insert fragment of positive prey plasmids were amplified by PCR and sequenced and then blasted against NCBI databases to identify the corresponding bovine genes. The predicted protein function of the identified genes was analyzed using Gene Ontology^1^, UniProt database^2^, and STRING^3^.

### Sub-Cellular Localization of TaCyp1 in TaNM1 Cells

The coding sequence of TaCyp1 was amplified from the cDNA of TaNM1 cells and cloned into *BamHI* and *XhoI* sites in pET30a using oligonucleotides: forward, CGC GGATCCATGTTAAAATTCTACAATCAACC, Reverse, CCG CTCGAGTCACAATAATTCTCCACAGTCC.

Subsequently, *E. coli* strain BL21DE3 (TransGen, Beijing, China) transformed with pET30a-TaCyp1 plasmid were cultured under the condition of 0.5 mM IPTG at 37°C for 8 h and then ultrasonic lysed after repeated freezing and thawing. The supernatant was collected by centrifugation and the recombinant His-TaCyp1 (rTaCyP1) protein was purified using Ni-NTA Purification System (Invitrogen, United States), which was used in sub-cellular localization and GST-pull down experiments. To obtain positive sera against rTaCyp1 New Zealand white rabbits were immunized with 200 μg of rTaCyp1 protein, emulsified in equal volumes of complete (day 0), or incomplete (days 14 and 28) Freund’s adjuvant (Sigma, United States) at 14-day intervals. Ten days after the last immunization sera were collected and purified using NAbTM Protein G Spin Kit (Thermo Fisher, United States). Sera before immunization were collected as negative control, and serum against TaSP used as positive control.

To investigate the localization of TaCyp1 TaNM1 cells were plated on coverslips for 24 h, and then fixed in PBS 4% paraformaldehyde for 20 min at room temperature. Coverslips were rinsed in PBS and permeabilized with PBS 0.5% Triton-X-100 for 5 min and then blocked with PBS 3% bovine serum albumin (BSA) at 37°C for 30 min to prevent non-specific staining. Subsequently, the coverslips were incubated with positive sera against TaCyp1 or TaSP (1/100) in PBS 1% BSA for 1 h at 37°C. After washing in PBS, the coverslips were stained with Hoechst 33342 (1/2000, Invitrogen, United States) and goat anti-rabbit IgG (H+L) secondary antibody Alexa Fluor 488 (1/1000, Invitrogen, United States) in PBS 1% BSA for 1 h at 37°C. Finally, coverslips were washed in PBS and stained with Alexa Fluor^TM^ 594 Phalloidin in PBS 1% BSA for 30 min at 37°C (1/100, Invitrogen, United States). All coverslips were washed in PBS and transferred to slides to observe using confocal microscopy (Leica, Germany).

### GST-Pull Down

To further verify the interaction between TaCyp1 and prey proteins the coding sequences of prey proteins were amplified from cDNA of TaNM1 cells and cloned into pGEX-4T-1 plasmid. The recombinant pGEX-4T-1-prey plasmids were transformed and expressed in *E. coli* strain BL21 (TransGen, Beijing, China) and purified using glutathione-sepharose beads. Following protocols of Pierce^TM^ GST Protein Interaction Pull-Down Kit (Thermo Fisher Scientific, United States), beads coated with GST-tagged prey protein from 10 ml IPTG-induced *E. coli* culture were incubated with 100 μg His-TaCyp1 protein for 8 h at 4°C. Subsequently, beads were washed five times with washing buffer and eluted by 200 μL elution buffer. Anti-His tag (1/2000, Sigma, United States) or anti-GST tag mouse McAb (1/2000, Signal way Antibody, United States) were used as primary antibodies, by western blot detection. Meanwhile, GST and His tag proteins were expressed under the same conditions.

### BW720c Treatment and siRNA Transfection

For drug-induced parasite death TaNM1 cells were treated with BW720C at 200 ng/mL for 72 h (Sigma, United States), and as a negative control were treated with an equal volume of DMSO. RNAi experiments were performed in six-well plates. 200 pm negative control or siRNA against target gene were transfected into TaNM1 cells using 10 μL lipofectamine 2000 reagent (Invitrogen, United States) and cells were collected in 36 h after siRNA transfection. All collected cells were used to extract total RNA or protein for RT-qPCR or western blot detection.

### RT-qPCR

For all samples, total RNA was extracted using TRIzol reagent and the cDNA was synthesized using Prime-Script^TM^ RT reagent with gDNA Eraser kit (TaKaRa, Dalian, China). Quantitative PCR was performed using SYBR^TM^ Premix Ex Taq^TM^ II (Tli RNaseH Plus) (TaKaRa, Dalian, China) according to the following primers: TaCyp1, forward, TGGTTAAGGCAGT TGAAG, reverse, AATAATTCTCCACAGTCCTC; NF-κB1 (NM_001076409.1), forward, AAGAACAAGAAGTCCTACC, reverse, GACCAACTGAACAATAACC; NF-κB2 (NM_001102101.1), forward, GAGGATGATGAGAATGGAT, reverse, GAACACAATGGCATACTG; β-actin (Marsolier et al., 2015), forward, GGCATCCTGACCCTCAAGTA, reverse, CACACGGAGCTCGTTGTAGA. Relative gene expression comparisons were performed using ΔΔCT method and β-actin gene was used for stably expressed housekeeping gene.

### Western Blot

For all samples, cells were lysed on ice for 30 min with Western and IP buffer (Beyotime, China) and cytoplasmic proteins in supernatant were collected by centrifugation at 10,000 *g* for 10 min. Subsequently, cytoplasmic proteins were separated by 12% SDS–PAGE gel and transferred to PVDF membranes (Millipore, United States). Membranes were blocked in TBS containing 5% BSA and 0.05% Tween-20 for 1 h at room temperature. Incubations with diluted primary antibodies in TBS 0.05% Tween-20 were performed at 4°C overnight. After 2 h incubation with an anti-rabbit or anti-mouse peroxidase-conjugated antibody (1/5000, Sigma, United States) at room temperature, proteins were detected by chemiluminescence (Thermo Fisher Scientific, United States). These antibodies were used: rabbit anti-NK-κB1 p105 (1/1000, Cell Signaling, United States), rabbit anti-phospho-IκBα/β (1/1000, Bioss Antibodies, China), mouse anti-NF-κB p52 and mouse anti-β-actin (1/1000, Santa Cruz, United States).

## Results

### TaCyp1 Expressed in Y2HGold Cells Has an Apparent Molecular Mass of 45 kDa

Total proteins were extracted from Y2HGold cells transformed with pGBKT7-TaCyp1 or pGBKT7-53 plasmid and detected with an anti-Myc tag mouse mAb. The size of TaCyp1 and positive control were 45 and 57 kDa, respectively, which is consistent with their calculated molecular mass (Figure 1C).

### TaCyp1 Bait Protein Has No Auto-Activation or Toxicity

The results of auto-activation showed the colonies containing pGBKT7 or pGBKT7- TaCyp1 plasmid were white on SD/-Trp/X plates, but blue on DDO/X/A plates for positive control, indicating that the TaCyp1 bait plasmid has no auto-activation activity (Figure 1B). Moreover, the size of the colonies containing bait plasmid was similar to that containing the pGBKT7 plasmid, indicating that the TaCyp1 bait plasmid has no toxicity in Y2HGold cells and that it could be used in the yeast-two-hybrid screen.

### Two Host Proteins Interact With TaCyp1

**FIGURE 2:**
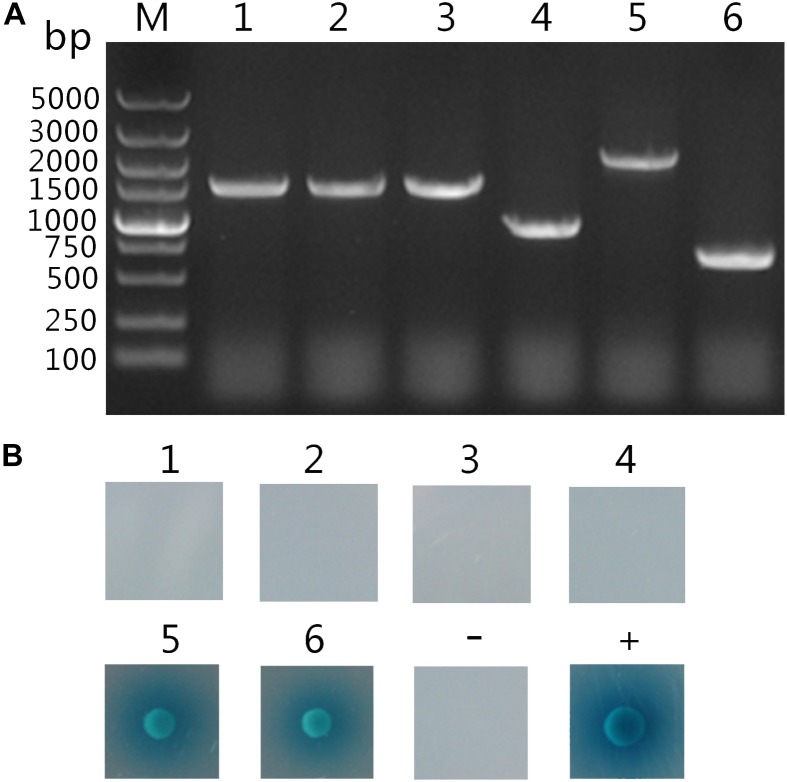
Analysis of putatively positive colonies. **(A)** Analysis of PCR products of the inserts on putatively positive prey plasmids. M: DNA 5000 maker; Lane 1–6: PCR amplification products of the inserts on the six putatively positive hits. **(B)** Confirmation of putative hits. The pGBKT7-TaCyp1 bait plasmid was transformed with each of the six putatively positive prey plasmids (number 1 to 6) into Y2HGold cells. Meanwhile pGADT7-T and pGBKT7-Lam plasmids were co-transformed into Y2HGold cells for negative control, whereas pGADT7-T and pGBKT7-53 were co-transformed into Y2HGold cells for positive control. All co-transformants were grown on QDO/X/A plates, and positive interaction was indicated by the presence of blue colonies.

Following screening on higher stringency QDO/X/A plates, six blue colonies were finally obtained, which were likely to be positive hits. After plasmid extraction prey plasmids were rescued by transforming *E. coli* DH5α cells. Subsequently, the inserts were amplified by PCR using primers of pGADT7-F/R and their size indicated by gel electrophoresis (Figure 2A). To eliminate false positive hits, each of these six prey plasmids was co-transformed with pGBKT7- TaCyp1 into Y2HGold cells and the co-transformants were grown on QDO/X/A plates. Only two co-transformants gave blue colonies growth (Figure 2B). As a control, co-transformants containing pGKBT7 and each prey plasmid gave no colonies on QDO/X/A plates (data not shown). So these two host proteins were considered to interact with TaCyp1.

The two fragments displayed 99% similarity with *Bos taurus* mediator complex subunit 21 (MED21, accession no.: NM_001038566.1) and *Bubalus bubalis* SEC31 homolog A transcript variant X8 (SEC31A, accession no.: XM_006074437.1), respectively. The two fragments encoded full-length of MED21 and 523 residues from the C terminus of SEC31A, respectively. Interestingly, functional prediction suggested MED21 could be a mediator of RNA polymerase II transcription subunit 21 and component of a coactivator involved in regulating transcription of nearly all RNA polymerase II-dependent genes. In general, MED21 is recruited to promoters by direct interactions with regulatory proteins and serves as a scaffold for the assembly of a functional preinitiation complex with RNA polymerase II and the general transcription factors. SEC31A is a component of the coat protein complex II (COPII), which is involved in transporting secreted and membrane proteins out of the endoplasmic reticulum. Through direct interaction with Sar1 and SEC23, the SEC13-SEC31 complex polymerizes into a polyhedral cage structure to form the functional outer coat that drives vesicle formation (Stagg et al., 2006; Bi et al., 2007; Fath et al., 2007).

### TaCyp1 Is Mainly Distributed Around *T. annulata* Schizonts

**FIGURE 3:**
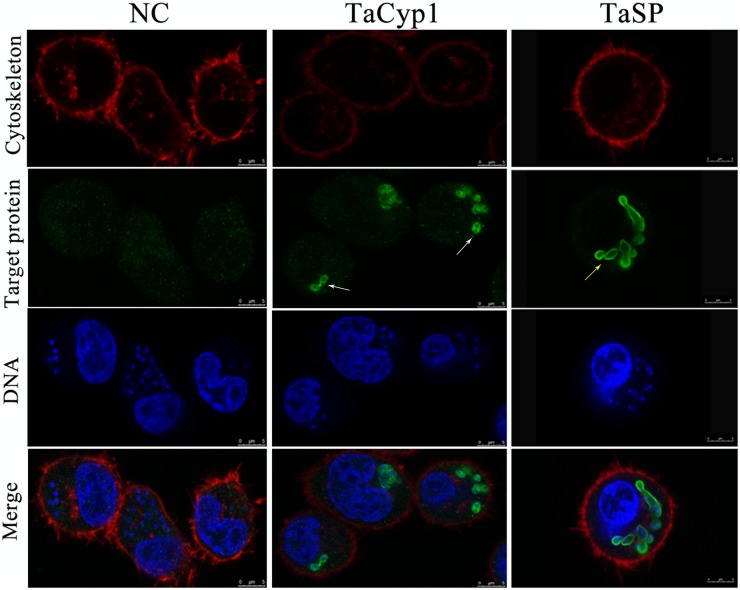
Sub-cellular localization of target proteins. NC stained with negative serum was no green fluorescence, and TaSP was observed with green fluorescence on membrane of *T. annulata* shizonts as positive control (yellow arrow). TaCyp1 was mainly distributed around membrane of *T. annulata* shizonts (white arrow). Cell cytoskeleton stained with Alexa Fluor^TM^ 594 Phalloidin and DNA stained with Hoechst 33342 were observed with red fluorescence and blue fluorescence, respectively, and target proteins stained with purified sera and Alexa Fluor 488 were observed with green fluorescence. The merge were fused images under the above three conditions.

To reduce non-specific staining all sera were purified using NAbTM Protein G Spin Kit. As a positive control a serum against TaSP was used and gave green fluorescence associated with membrane of *T. annulata* schizonts, consistent with TaSP being a membrane protein (Seitzer et al., 2010). No green fluorescence was observed with samples stained with negative serum. For samples stained with serum against TaCyp1 green fluorescence was mainly observed around *T. annulata* schizonts (Figure 3). DNA stained with Hoechst 33342 (blue) and cytoskeleton stained with Alexa Fluor^TM^ 594 Phalloidin (red) were observed in all samples. The images indicate that in TaNM1 cells TaCyp1 is mainly distributed around *T. annulata* schizonts, a localization providing an opportunity to interact with host proteins.

### MED21 Interacts *in vitro* Directly With TaCyp1

**FIGURE 4:**
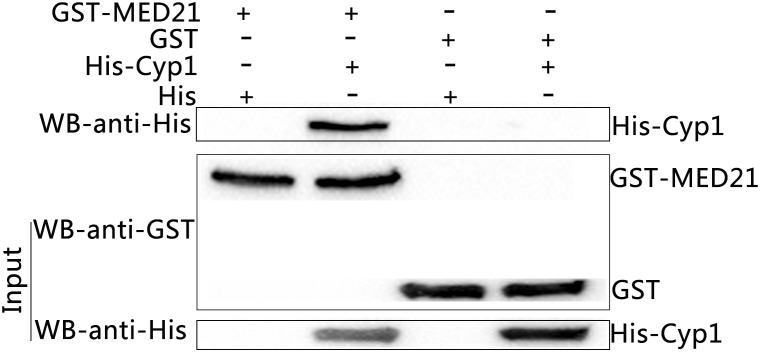
Western blot analysis of GST-pull down experiments. Input were the mixed supernatants including GST-MED21+His tag protein, GST-MED21+His-Cyp1, GST tag protein+ His tag protein, GST tag protein+ His-Cyp1 from left to right, and identified by western blot using anti- His or GST tag antibody. The eluted protein complexes were identified by western blot with anti-His tag protein antibody.

The predicted sequences of the prey proteins identified them to be MED21 and SEC31A and so both inserts were cloned into the *BamHI* and *XhoI* restriction sites of pGEX-4T-1 p. The oligonucleotides used were: MED21, forward, CGCGGATCCAGGAACATGGCGGATCGGCT, reverse, CCGCTCGAGTGAGTCTGGAAGAGACTGG; SEC31A, forward, CGCGGATCCAAACTAA TTGCATGTTGGAC, reverse, CCGCTCGAGGACACCCAGCTTGTTGGCCT. Subsequently, GST-MED21 or GST-SEC31A recombinant proteins were expressed in *E. coli* strain BL21 (TransGen, Beijing, China). GST pull-downs showed GST tag or GST-MED21 recombinant protein did not bind to His tag protein. Meanwhile, His-TaCyp1 protein also had no binding to GST tag protein *in vitro*, but in contrast GST-MED21 bound to His-TaCyp1 indicating a direct interaction between TaCyp1 and MED21 (Figure 4). For the GST-SEC31A recombinant protein no binding was observed between GST-SEC31A and His-TaCyp1 proteins (data not shown). So we focused on the interaction of TaCyp1 with the host protein MED21.

### Knock Down of MED21 Results in a Significant Decrease in NF-κB1/κB2 mRNA Expression

**FIGURE 5:**
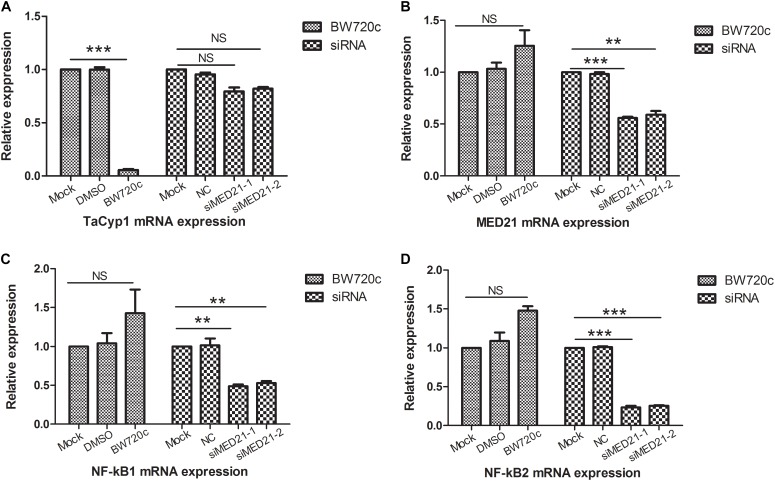
mRNA expression analysis of TaCyp1, MED21, NF-κB1, and NF-κB2. mRNA expressions of TaCyp1, MED21, NF-κB1, and NF-κB2 in TaNM1 cells were detected after BW720c treatment and MED21 RNAi experiments **(A–D)**. In BW720c treatment experiment, mock group was added with PBS, NC group was added with DMSO; positive group was added with equal volume of BW720c; in MED21 RNAi experiment, mock group was with no RNA transfection, NC group was transfected with NC siRNA, positive group was transfected with siRNA. ^∗∗^*P* < 0.01; ^∗∗∗^*P* < 0.001. NS, not significant.

Quantitative PCR primers of MED21A was used to detect MED21 mRNA expression: forward, ATATTCAGACAGCCAT TA, reverse, GAATCTATCAGAACATCAAT. Meanwhile the NC and MED21 siRNA were used in RNAi experiment: NC, sense, UUCUCCGAACGUGUCACGUTT, antisense, ACGUGACACGUUCGGAGAATT; siMED21-1, sense, GGACGCUGUGAACUCGCUUTT, antisense, AAGCGAGUUCACAGCGUCCTT; siMED21-2, sense, CCUCCUGCCUCCUUUAGUATT, antisense, UACUAAAGGAGGCAGGAGGTT. To investigate the potential role of MED21 in *Theileria*-infected cells, we used siRNA to deplete MED21 by siRNA. After siMED21 RNAi, MED21 mRNA expression was significantly reduced (Figure 5B). Because MED21 is a component of the RNA polymerase II transcriptional subunit, we hypothesized that knock down of MED21 might lead to a reduction in NF-κB1/2 mRNA expression. This was indeed the case (Figures 5C,D). We also analyzed TaCyp1, MED21, and NF-κB1/2 mRNA levels following BW720c treatment. As expected, we found that TaCyp1 levels significantly decreased following killing of the parasite (Figure 5A). In contrast, there was no significant change in MED21 and NF-κB1/2 mRNA expression following treatment.

### MED21 Does Not Affect NF-κB Signaling

**FIGURE 6:**
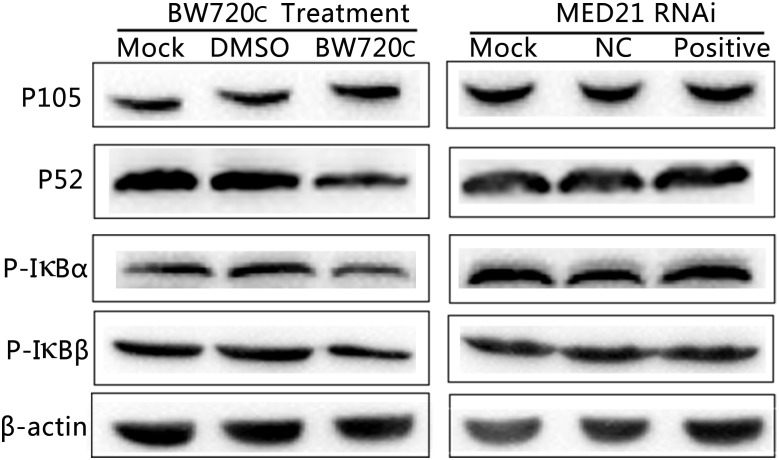
Western blot analysis of cytoplasmic proteins in TaNM1 cells after BW720c treatment and MED21 RNAi. After BW720c treatment and MED21 RNAi, protein expressions of P105, P52, and Phosphorylated IκBα/β proteins were detected using anti-NF-κB1 P105, anti-NF-κB2 P52, anti-phospho IκBα and IκBβ antibodies. As reference protein, β-actin were detected using mouse anti-β-actin antibody.

After BW720c treatment there was no significant increase in expression of precursor protein P105, but an obvious decrease in that of P52 and the amount of phosphorylated IκBα/β, indicating a decrease in NF-κB signaling. In contrast, there was no obvious change on P105, P52, and phosphorylated IκBα/β protein expressions after MED21 siRNA interference (Figure 6). The results showed that the absence of *T. annulata* schizont had a significant influence on the activity of NF-κB signaling pathway. However, knock down of MED21 had no obvious impact on NF-κB signaling.

## Discussion

As a protein family cyclophilins have been implicated in protein folding, as molecular chaperones, in trafficking, signaling cell activation (Nigro et al., 2013). In this study, A 684 bp fragment of TaCyp1 gene was successfully amplified from the cDNA of TaNM1 cells and its sequence to have 88% identity with *T. parva* cyclophilin1 (accession no.: XP_765771.1). The predicted protein structure analyzed by online software SMART showed TaCyp1 contained a signal peptide (aa1-22) and a Pro-isomerase domain (aa65-226), which was classified as family CypA by CD-Search (Marchler-Bauer and Bryant, 2004). Western blot results showed TaCyp1 was correctly expressed in Y2HGold cells and when used as bait to screen for host cell interacting proteins by yeast-two-hybrid system identified MED21 and SEC31A. However, only MED21 was confirmed to interact with TaCyp1 in GST-pull down experiments. TaCyp1 displayed a similar distribution to TaSP by being associated with the surface of schizont where it has the opportunity to interact with host cell MED21.

MED21 plays an integral role in activation of RNA polymerase II (Pol II) transcription. In general in human cells, the mediator head or middle module includes seven subunits (MED6, -8, -11, -17, -18, -20, and -22) or eight subunits (MED1, -4, -7, -9, -19, -10, -21, and -31) (Sato et al., 2016). Among all subunits, MED21 is the most conserved and necessary for cell viability in yeast and mice (Hengartner et al., 1995; Tudor et al., 1999; Bourbon, 2008). Sato et al. (2016) found transcription of an NF-κB-driven reporter gene was obviously inhibited after MED21 RNAi. Moreover, the first 15 amino acids of MED21 was lethal to *Saccharomyces cerevisiae* (Gromoller and Lehming, 2000). NF-κB signaling pathway is activated in *T. annulat*a-infected cells (Heussler et al., 2006) and NF-κB subunits usually exist in the form of homodimers or heterodimers, such as P50/RelA, P52/RelB, P50/P50, and P52/P52, among of which P50/RelA heterodimer is most widely distributed. Through hydrolysis of ATP dependent protease, precursor protein P105 or P100 can be hydrolyzed to be P50 or P52, respectively. In general, the activity of NF-κB is regulated by members of IκB family (Lenardo et al., 1987). To explore the influence of MED21 on NF-κB signaling in TaNM1 cells, BW720c-induced parasite death and MED21 RNAi knock down were performed. Protein expression of P52 and phosphorylated IκBα/β levels were significantly decreased after BW720c treatment, and similar results have been previously reported by Schmuckli-Maurer et al. (2010). However, we observed no obvious change in P105 protein levels or NF-κB1/2 mRNA expression after BW720c treatment. In contrast, MED21 mRNA expression was significantly decreased after siMED21 RNAi. As a component of regulatory subunit of RNA polymerase II transcription, MED21 is normally involved in regulating the transcription of almost all polymerase RNAII dependent genes. So knock down of MED21 should also result in a significant decrease on NF-κB1/2 mRNA expressions, but we could observe no significant change on P105 and P52 levels and no change in the phosphorylation status of IκBα/β. This implies that loss of MED21 could be compensated by other molecules, but understanding why there was no change in NF-κB1/2 levels and IκBα/βphosphorylation following MED21 knock down will required further work. Clearly, absence of the *T. annulata* schizont has a significant influence on the activity of NF-κB signaling and even though knock down of MED21 led to a significant decrease in mRNA expressions of NF-κB1/2, there was no obvious influence on the NF-κB signaling pathway.

In this study it describes that the host protein MED21 interacts with TaCyp1 providing insight into the function of bovine MED21, and its interaction with *T. annulata* in infected leukocytes. In future experiments new host proteins will be identified using yeast two hybrid screening and gene editing technologies such as CRISPR-Cas9 can be used to uncover the consequences of molecular interactions between *T. annulata* schizonts and their bovine leukocyte hosts.

## Conclusion

In this study, A 684 bp fragment of the TaCyp1 gene was successfully amplified from the cDNA of TaNM1 cells and correctly expressed at the expected size of 45 kDa in Y2HGold cells. After yeast-two-hybrid screening two host proteins MED21 and SEC31A were identified as putative interaction molecules. Following GST-Pull down only MED21 was confirmed to interact with TaCyp1 *in vitro.* The distribution of TaCyp1 around *T. annulata* schizonts is consistent with an interaction with MED21. Classically as component of mediator complex MED21 regulates the transcription of nearly all RNA polymerase II-dependent genes. To explore its influence on NF-κB signaling in *T. annulata*-infected leukocytes MED21 expression was knocked down by RNAi and as a positive control parasite death was induced by BW720c treatment. Drug-induced elimination of *T. annulata* schizonts had a significant influence on activity of NF-κB signaling pathway. However, even though knock down of MED21 resulted in a significant decrease in mRNA expression of NF-κB1/2, no obvious influence on the NF-κB signaling pathway could be observed. Why there are no observable change in protein expressions of NF-κB1/2 and phosphorylation of IκBα/β after RNAi knock down of MED21 requires further study.

## Ethics Statement

The study was approved by the Animal Ethics Committee of Lanzhou Veterinary Research Institute, Chinese Academy of Agricultural Sciences.

## Author Contributions

JiL, HY, and GG designed the study and critically revised the manuscript. SZ performed all of the experiments and drafted the manuscript with the help of JuL. AL and YL contributed to the revision of the manuscript.

## Conflict of Interest Statement

The authors declare that the research was conducted in the absence of any commercial or financial relationships that could be construed as a potential conflict of interest.

## Footnotes

1 http://amigo.geneontology.org/amigo

2 http://www.uniprot.org/

3 http://string.embl-heidelberg.de/
